# Heterogeneous treatment effects of dexamethasone 12 mg versus 6 mg in patients with COVID‐19 and severe hypoxaemia—Post hoc exploratory analyses of the COVID STEROID 2 trial

**DOI:** 10.1111/aas.14167

**Published:** 2022-11-08

**Authors:** Anders Granholm, Marie Warrer Munch, Nina Andersen‐Ranberg, Sheila Nainan Myatra, Bharath Kumar Tirupakuzhi Vijayaraghavan, Balasubramanian Venkatesh, Vivekanand Jha, Rebecka Rubenson Wahlin, Stephan M. Jakob, Luca Cioccari, Morten Hylander Møller, Anders Perner

**Affiliations:** ^1^ Department of Intensive Care Rigshospitalet—Copenhagen University Hospital Copenhagen Denmark; ^2^ Collaboration for Research in Intensive Care Copenhagen Denmark; ^3^ Department of Anaesthesiology and Intensive Care Medicine Zealand University Hospital Køge Denmark; ^4^ Department of Anaesthesia, Critical Care and Pain Tata Memorial Hospital, Homi Bhabha National Institute Mumbai India; ^5^ Department of Critical Care Apollo Hospitals Chennai India; ^6^ Chennai Critical Care Consultants Chennai India; ^7^ The George Institute for Global Health New Delhi India; ^8^ The George Institute for Global Health, University of New South Wales Sydney Australia; ^9^ Prasanna School of Public Health Manipal Academy of Higher Education Manipal India; ^10^ School of Public Health Imperial College London London UK; ^11^ Department of Clinical Science and Education, Södersjukhuset Karolinska Institutet Stockholm Sweden; ^12^ Department of Intensive Care Medicine, Inselspital Bern University Hospital, University of Bern Bern Switzerland; ^13^ Department of Intensive Care Medicine Kantonsspital Aarau Aarau Switzerland

**Keywords:** corticosteroids, COVID‐19, critical illness, days alive without life support, hypoxaemia, mortality

## Abstract

**Background:**

Corticosteroids improve outcomes in patients with severe COVID‐19. In the COVID STEROID 2 randomised clinical trial, we found high probabilities of benefit with dexamethasone 12 versus 6 mg daily. While no statistically significant heterogeneity in treatment effects (HTE) was found in the conventional, dichotomous subgroup analyses, these analyses have limitations, and HTE could still exist.

**Methods:**

We assessed whether HTE was present for days alive without life support and mortality at Day 90 in the trial according to baseline age, weight, number of comorbidities, category of respiratory failure (type of respiratory support system and oxygen requirements) and predicted risk of mortality using an internal prediction model. We used flexible models for continuous variables and logistic regressions for categorical variables without dichotomisation of the baseline variables of interest. HTE was assessed both visually and with *p* and *S* values from likelihood ratio tests.

**Results:**

There was no strong evidence for substantial HTE on either outcome according to any of the baseline variables assessed with all *p* values >.37 (and all *S* values <1.43) in the planned analyses and no convincingly strong visual indications of HTE.

**Conclusions:**

We found no strong evidence for HTE with 12 versus 6 mg dexamethasone daily on days alive without life support or mortality at Day 90 in patients with COVID‐19 and severe hypoxaemia, although these results cannot rule out HTE either.

**Editorial Comment**

In this post hoc explorative sub‐study of the COVID STEROID 2 trial, no strong evidence for substantial heterogeneity in treatment effects was found. The authors included the *S*‐value for the interpretation of probabilities, which may be a more understandable measurement compared to the standard *p*‐value, both for clinicians and researchers.

## 1 INTRODUCTION

Coronavirus disease 2019 (COVID‐19) may cause critical illness and high mortality rates due to severe pulmonary inflammation and hypoxaemia.^1^ Anti‐inflammatory treatment, including corticosteroids, reduces mortality and is recommended for patients with severe and critical COVID‐19.^2^, ^3^

In the COVID STEROID 2 randomised controlled trial, we assessed 12 versus 6 mg of dexamethasone for patients with COVID‐19 and severe hypoxaemia and found high probabilities of benefit from the higher dose for all outcomes assessed up until Day 90; long‐term outcomes were similarly mostly compatible with benefit, albeit not reaching the thresholds for statistical significance.^4^, ^5^, ^6^

Despite probable overall benefits with a higher dose, heterogeneous treatment effects (HTE)^7^, ^8^, ^9^ in different patient groups may be present, as has been suggested in previous critical care trials.^9^, ^10^, ^11^, ^12^ In the conventional, dichotomous subgroup analyses, no statistically significant HTE was found for the primary outcome (days alive without life support at Day 28) according to several baseline characteristics (including age and weight dichotomised at 70 years and 80 kg, respectively).^4^, ^13^ However, conventional subgroup analyses are at risk of type 2 errors as trials are generally only powered for the primary analysis,^8^, ^9^ dichotomisation of continuous variables further decreases power,^14^ and focus on individual variables may not correspond well with the clinical reality, where risk and treatment decisions are affected by the combinations of multiple factors.^8^, ^9^

In this post hoc exploratory sub‐study of the COVID STEROID 2 trial, we aimed to assess whether HTE with two different doses of dexamethasone was present for the number of days alive without life support and mortality at Day 90 according to four baseline characteristics (age, weight, category of respiratory failure and number of comorbidities) and the predicted risk of 90‐day mortality, all assessed without dichotomisation of the variables included or the conclusions.^15^

## 2 METHODS

These post hoc exploratory analyses of HTE in the COVID STEROID 2 trial were conducted according to a statistical analysis plan, which was written after the pre‐planned analyses of the trial were reported,^4^, ^5^, ^6^ but before any of the analyses reported in this manuscript were conducted.^15^ This manuscript was prepared according to the Strengthening the Reporting of Observational Studies in Epidemiology (STRO) checklist^16^ (supplement).

### 2.1 The COVID STEROID 2 trial

The COVID STEROID 2 trial was an investigator‐initiated, international, parallel‐group, stratified, blinded (including patients, clinicians, investigators and outcome assessors) randomised clinical trial, approved by the regulatory authorities and ethics committees in all participating countries.^4^, ^17^ One thousand adult patients hospitalised with COVID‐19 and severe hypoxaemia (≥10 L oxygen/min, use of non‐invasive ventilation [NIV], continuous use of continuous positive airway pressure [CPAP] or invasive mechanical ventilation) were enrolled at 31 sites in 26 hospitals in Denmark, India, Sweden and Switzerland between 27 August 2020 and 20 May 2021.^4^ Patients were primarily excluded due to previous use of systemic corticosteroids for COVID‐19 in doses >6 mg for ≥5 days, unobtainable consent, and use of higher‐dose steroids for other indications than COVID‐19.^4^, ^17^ Patients were randomised 1:1 to dexamethasone 12 or 6 mg intravenously once daily for up to 10 days. Additional details are provided in the primary protocol and trial report.^4^, ^17^

### 2.2 Outcomes and patients assessed

In this sub‐study, we assessed the following two outcomes at 90 days:

1. Days alive without life support (including invasive mechanical ventilation, circulatory support and kidney replacement therapy; the actual number of days was used without assigning dead patients the worst possible value).
2. Mortality.

Of note, the primary outcome in the COVID STEROID 2 trial was days alive without life support after 28 days of follow‐up for logistical/ethical reasons due to the urgency of the pandemic.^4^, ^17^

Both outcomes were assessed in the complete intention‐to‐treat (ITT) population (*n* = 982 after exclusion of patients without consent for the use of their data^4^); no formal sample size calculation was conducted for this post hoc study.

### 2.3 Statistical analyses

#### 2.3.1 Descriptive data

We present descriptive data for all baseline and outcome variables assessed in this study in both treatment groups with continuous variables presented as medians with interquartile ranges (IQRs) and full ranges, and binary and categorical variables presented as numbers with percentages.

#### 2.3.2 Heterogeneity in treatment effects

We assessed HTE using frequentist analyses without adjustment according to the following four baseline characteristics:

1. Age (years)
2. Weight (kg)
3. Category of respiratory failure on a 1–6‐point scale defined as follows:
  1. Open system, low oxygen flow (oxygen flow rate ≤ median oxygen flow rate in all patients on open systems).
  2. Open system, high oxygen flow (oxygen flow rate > median oxygen flow rate).
  3. NIV/CPAP, low fraction of inspired oxygen (FiO_2_; FiO_2_ ≤ median FiO_2_ in all patients on closed systems).
  4. NIV/CPAP, high FiO_2_ (FiO_2_ > median FiO_2_).
  5. Invasive mechanical ventilation, low FiO_2_ (defined as above).
  6. Invasive mechanical ventilation, high FiO_2_ (defined as above).
4. Number of comorbidities (diabetes mellitus, ischemic heart disease or heart failure, chronic obstructive pulmonary disease or immunosuppression within 3 months prior to randomisation) on a 1–4‐point scale with patients with three or four comorbidities analysed in the same category as only one patient had all four comorbidities.

In addition, we assessed HTE according to the baseline predicted risk of 90‐day mortality using an internal prediction model developed in the control group (6 mg) as described below.

#### 2.3.3 Analytical strategy

We assessed HTE on the continuous scale for age, weight and predicted mortality risk using generalised additive models (linear/logistic regressions, respectively, for the two outcomes) with cubic regression splines, fixed degrees of freedom and five knots at the 5, 27.5, 50, 77.5 and 95 percentiles.^15^, ^18^ Primary models included treatment and a smooth‐by‐treatment allocation separately for each characteristic assessed with likelihood ratio tests used to assess the treatment‐by‐baseline variable‐interaction by comparing the full models to models only including treatment and a smooth transformation of the variable of interest not stratified by treatment allocation.

HTE according to the category of respiratory failure and the number of comorbidities was assessed by conventional linear/logistic regression models, including treatment, the baseline variable of interest (as a categorical variable) and an interaction term; likelihood ratio tests were used to assess interactions similarly as for the generalised additive models. Of note, this was not specified in the statistical analysis plan,^15^ but necessary as generalised additive models could not be used due to a few distinct values for these two baseline variables.

Results are presented graphically using predicted mean outcome values with 95% confidence intervals (CIs) in each treatment group according to values of the baseline variable in question, supplemented with plots illustrating the absolute differences between groups with 95% CIs.

The *p* values from the likelihood ratio tests are presented; results were not dichotomised according to significance thresholds but interpreted as continuous measures of evidence, with results interpreted cautiously and only as hypothesis generating due to their post hoc nature.^15^ To supplement the interpretation, we converted *p* values to *S* values (*S*‐value = −log_2_[*p*‐value]).^19^ In brief, *S* values measure how ‘surprising’ the observed results are assuming that there is truly no difference on an interpretable scale; *S* values thus correspond to the chance of getting all heads in *S* consecutive fair coin tosses.^19^

#### 2.3.4 Internal prediction model

We developed an internal prediction model^15^, ^20^ for 90‐day mortality using the control group only. The following baseline variables were entered into a logistic regression model: age (years), weight (kg), type of respiratory support (open system [reference], NIV/CPAP, or invasive mechanical ventilation), diabetes mellitus, ischaemic heart disease or heart failure, chronic obstructive pulmonary disease, immunosuppression within 3 months prior to randomisation, baseline lactate concentration (mmol/L), use of vasopressors or inotropes at baseline and limitations in care (e.g., cardio‐pulmonary resuscitation, life support) at baseline.

Continuous variables were modelled using multivariable fractional polynomials^21^ with the best‐fitting second‐degree fractional polynomial transformation of each continuous variable used. Apparent internal performance was assessed using the area under the receiver operating characteristics curve (AUROC; assessing discrimination, i.e., the chance that a patient with the event in question has a higher predicted risk than one without, with 0.5 being equal to chance and 1.0 corresponding to perfect discrimination); and calibration was assessed using calibration plots (with predicted/observed mortality presented in tenths, using a *loess* smoother and a linear regression on the predicted/observed values).^22^ We present predicted risks in both treatment groups and the full resulting model.

#### 2.3.5 Missing data

The amounts of missing data for the outcome variables and most baseline variables assessed were negligible except for lactate levels;^4^ in total, 1.4%–1.5% of the ITT population had missing data for the outcomes assessed, 1.3% of the ITT population had missing FiO_2_‐data and 10.8% had missing lactate values. All analyses except the prediction model‐based analyses were thus conducted using complete cases only, while the prediction model‐based analyses were conducted using multiply imputed datasets.^23^ We generated 25 imputed datasets separately in each group using the predictive mean matching and logistic regression methods, including all variables mentioned above and the country of enrollment.^15^, ^24^ Knot positions and optimal fractional polynomial transformations were calculated using a single imputed dataset (with prediction imputation not accounting for between‐imputation uncertainty and without imputation of missing outcome data), followed by fitting all final models on the 25 multiply imputed datasets. Predicted values were combined using Rubin's rules; *p* values from the likelihood ratio test for the model comparisons were pooled after transformation to the *Z*‐scale followed by back‐transformation.^25^ For all plots, predicted values were calculated with 95% CIs for 100 distinct values equally spaced between the minimum/maximum values displayed.

#### 2.3.6 Software

Analyses were conducted using R (R Core Team, R Foundation for Statistical Computing, Vienna, Austria) v. 4.1.0 with the *mgcv*, *mice*, *mfp* and *Tidyverse* packages.

#### 2.3.7 Additional analyses added during peer review

During the peer review process, additional descriptive baseline data and analyses not outlined in the statistical analysis plan^15^ were added. These were analyses on the continuous scale according to PaO_2_/FiO_2_‐ratios in patients on closed systems only and according to PaO_2_/oxygen flow‐ratios in patients on open systems only. These analyses were conducted in complete cases only.

## 3 RESULTS

Descriptive baseline and outcome data for the 982 patients in the ITT population are presented in Table 1. Treatment groups were largely similar, although the number of comorbidities was slightly lower in the 12‐mg group, mostly due to the lower presence of diabetes. As previously reported,^4^ the 12‐mg group had a higher median number of days alive without life support and lower mortality at day 90, although smaller effects in the opposite directions could not be excluded.

**TABLE 1:** Descriptive baseline and outcome data

Variable	12 mg (*n* = 497)	6 mg (*n* = 485)
Baseline variables		
Country of inclusion		
Denmark	251 (50.5%)	234 (48.2%)
India	182 (36.6%)	187 (38.6%)
Sweden	40 (8.0%)	39 (8.0%)
Switzerland	24 (4.8%)	25 (5.2%)
Age (years)	65 (56–74) [22–88]	64 (54–72) [22–90]
Weight (kg)	80 (68–96) [45–198]	80 (68–95) [42–164]
Type of respiratory support		
Open system	272 (54.7%)	258 (53.2%)
NIV/CPAP	118 (23.7%)	128 (26.4%)
Invasive mechanical ventilation	107 (21.5%)	99 (20.4%)
Respiratory failure category (presented as numerical values)^a^	2 (1–4) [1–6]	2 (1–4) [1–6]
Respiratory failure category^a^		
1: Open system, low flow^b^	141 (28.7%)	126 (26.4%)
2: Open system, high flow^b^	131 (26.6%)	132 (27.7%)
3: NIV/CPAP, low FiO_2_ ^c^	69 (14.0%)	73 (15.3%)
4: NIV/CPAP, high FiO_2_ ^c^	45 (9.1%)	47 (9.9%)
5: Invasive mechanical ventilation, low FiO_2_ ^c^	64 (13.0%)	51 (10.7%)
6: Invasive mechanical ventilation, high FiO_2_ ^c^	42 (8.5%)	48 (10.1%)
Oxygen flow rate (L/min, in patients on open system only)	22 (15–40) [10–61]	24 (15–40) [10–70]
FiO_2_ (%, in patients on closed system only)^a^	60 (50–75) [25–100]	60 (45–80) [25–100]
Number of comorbidities	0 (0–1) [0–3]	1 (0–1) [0–4]
Number of comorbidities (categorical)		
0	270 (54.3%)	240 (49.5%)
1	164 (33.0%)	174 (35.9%)
2	54 (10.9%)	57 (11.8%)
3	9 (1.8%)	13 (2.7%)
4	0 (0.0%)	1 (0.2%)
Diabetes mellitus	135 (27.2%)	163 (33.6%)
Ischemic heart disease or heart failure	67 (13.5%)	69 (14.2%)
Chronic obstructive pulmonary disease	57 (11.5%)	56 (11.5%)
Immunosuppression within 3 months prior to randomisation	40 (8.0%)	43 (8.9%)
Lactate (mmol/L)^d^	1.6 (1.1–2.3) [0.3–16.7]	1.7 (1.2–2.3) [0.2–13.8]
Use of vasopressors or inotropes	81 (16.3%)	68 (14.0%)
Limitations in care (cardio‐pulmonary resuscitation or life‐support)	30 (6.0%)	25 (5.2%)
Predictions		
Predicted risk of mortality at day 90^e^ (%)	35.5 (22.2–52.9) [2.6–99.7]	34.6 (22.6–49.4) [3.1–98.3]
Outcome variables		
Days alive without life support at day 90^f^	84 (9–90) [0–90]	80 (6–90) [0–90]
Mortality at day 90^g^	157 (32.0%)	180 (37.7%)

*Note*: Descriptive baseline, outcome and predicted data according to the internal prediction model for all variables assessed. Some baseline and the outcome data have been presented previously elsewhere.^4^ Numeric data are presented as medians (interquartile ranges) [full ranges], whereas binary/categorical data are presented as numbers (%).

Abbreviations: CPAP, continuous positive airway pressure; NIV, non‐invasive ventilation.

^a^ A total of 13 patients on closed systems (1.3% of the full intention‐to‐treat population; 5 patients in the 12‐mg group and 8 patients in the 6‐mg group) could not be classified due to missing FiO_2_ values.

^b^ Low flow includes patients with oxygen flow values ≤ the median value in all patients on open systems (22 L/min), whereas high flow includes patients with values > this value.

^c^ Low FiO_2_ includes patients with values ≤ the median value in all patients on closed systems (60%), whereas high FiO_2_ includes patients with values > this value.

^d^ Lactate values were missing in 106 patients (10.8% of the intention‐to‐treat population; 57 patients in the 12‐mg group and 49 patients in the 6‐mg group).

^e^ Calculated using the stacked multiply imputed datasets; the mean predicted risk was 38.5% in the 12‐mg group and 37.6% in the 6‐mg group.

^f^ Days alive without life support at Day 90 were missing in 15 patients (1.5% of the intention‐to‐treat population; 8 patients in the 12 mg group and 7 patients in the 6 mg group).

^g^ Mortality at Day 90 values were missing in 14 patients (1.4% of the intention‐to‐treat population; 7 patients in the 12‐mg group and 7 patients in the 6‐mg group).

HTE according to treatment allocation and simple baseline characteristics.

**FIGURE 1:**
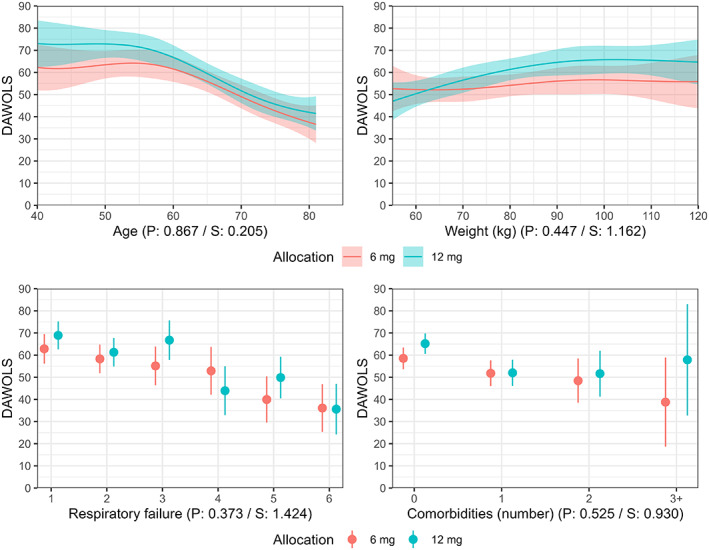
Days alive without life support at Day 90 according to treatment allocation and baseline characteristics. Expected mean number of days alive without life support (DAWOLS) with 95% confidence intervals according to four baseline variables (as described in methods section) according to the model fit. Predicted values and 95% confidence intervals are truncated at the lowest/highest possible values (0/90 days). The *p* and *S* values from the likelihood ratio tests assessing evidence in favour of heterogeneous treatment effects are displayed below each plot. For the continuous variables, predictions are only displayed for the central 90% of values in the data due to the large uncertainty at the extreme values with limited data. Figure S1 displays predicted values across all observed values in the datasets as specified in the statistical analysis plan.^15^

**FIGURE 2:**
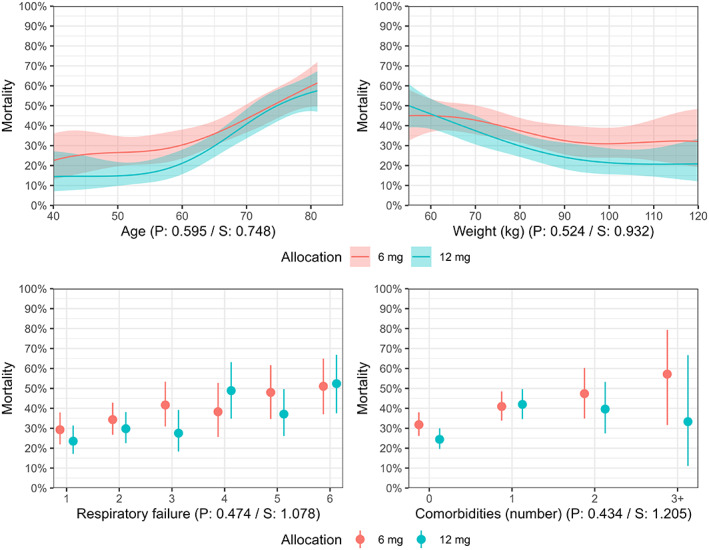
Mortality Day 90 according to treatment allocation and baseline characteristics. Expected mortality rates at Day 90 with 95% confidence intervals according to four baseline variables (as described in the methods section) according to the model fit. The *p* and *S* values from the likelihood ratio tests assessing evidence in favour of heterogeneous treatment effects are displayed below each plot. For the continuous variables, predictions are only displayed for the central 90% of values in the data due to the large uncertainty at the extreme values with limited data. Figure S2 displays predicted values across all observed values in the datasets as specified in the statistical analysis plan.^15^

Figures 1 and 2 (and Figures S1 and S2) present the expected mean number of days alive without life support and mortality at Day 90, respectively, according to treatment allocation and baseline characteristics. While outcomes appeared better with 12 mg dexamethasone in general,^4^, ^5^ there was no strong evidence of HTE according to any of the baseline characteristics on either outcome, with substantial overlap and parallelism between curves and all *p* values >.37 corresponding to all *S* values being <1.43. Visually and numerically, point estimates favoured 12 mg in patients weighing more and 6 mg in patients weighing less, but substantial uncertainty remains. Similarly, treatment effects appeared to be reversed (possibly favouring the lower dose) or neutral compared to the overall findings for patients on closed systems (NIV/CPAP or invasive mechanical ventilation) with the highest FiO_2_ values; as for weight, there was substantial overlap and uncertainty remains.

### 3.1 Internal prediction model and HTE

The performance of the internal prediction model (full model presented in the supplement) was adequate regarding both discrimination (AUROC 0.73, 95% CI 0.68–0.77) and calibration (Figure S3).

The median predicted risks of mortality were 35.5% (12 mg) versus 34.6% (6 mg) with mean predicted probabilities of 38.5% (12 mg) versus 37.6% (6 mg), respectively, while actual mortality rates were 32.0% (12 mg) versus 37.7% (6 mg) (Table 1).

**FIGURE 3:**
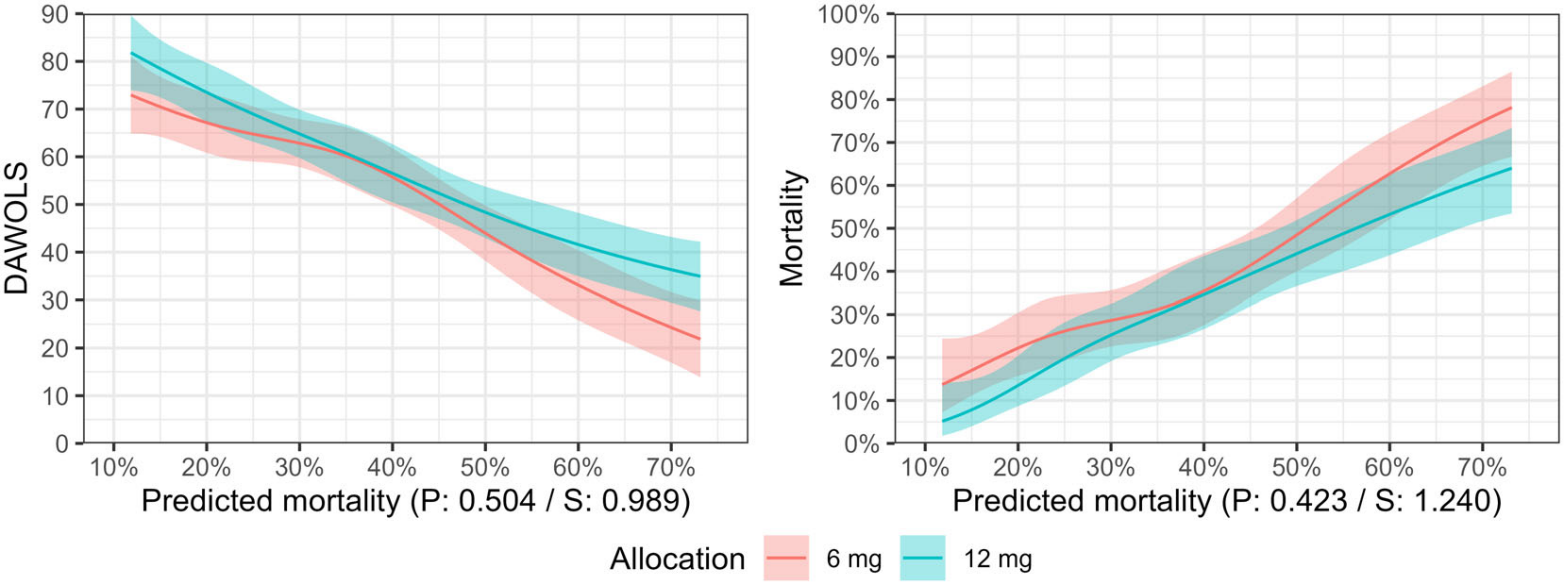
Days alive without life support and mortality Day 90 according to treatment allocation and predicted mortality risk. Expected mean number of days alive without life support (DAWOLS) and risk of mortality at Day 90 with 95% confidence intervals according to the predicted risks of mortality at Day 90 using the internal prediction model. For DAWOLS, predicted values and 95% confidence intervals are truncated at the lowest/highest possible values (0/90 days). *p* and *S* values from the likelihood ratio tests assessing evidence in favour of heterogeneous treatment effects are displayed below each plot. For these continuous variables, predictions are only displayed for the central 90% of values in the data due to the large uncertainty at the extreme values with limited data. Figure S4 displays predicted values across all observed values in the datasets as specified in the statistical analysis plan.^15^

The expected outcomes according to predicted mortality risk for both days alive without life support and mortality at Day 90 are presented in Figure 3 (and Figure S4). As for the simple baseline characteristics, there was no strong evidence for HTE with largely parallel and overlapping curves and *p* values of .50 for days alive without life support (*S*‐value 0.99) and .42 for mortality (*S*‐value 1.24).

### 3.2 Treatment effect differences

**FIGURE 4:**
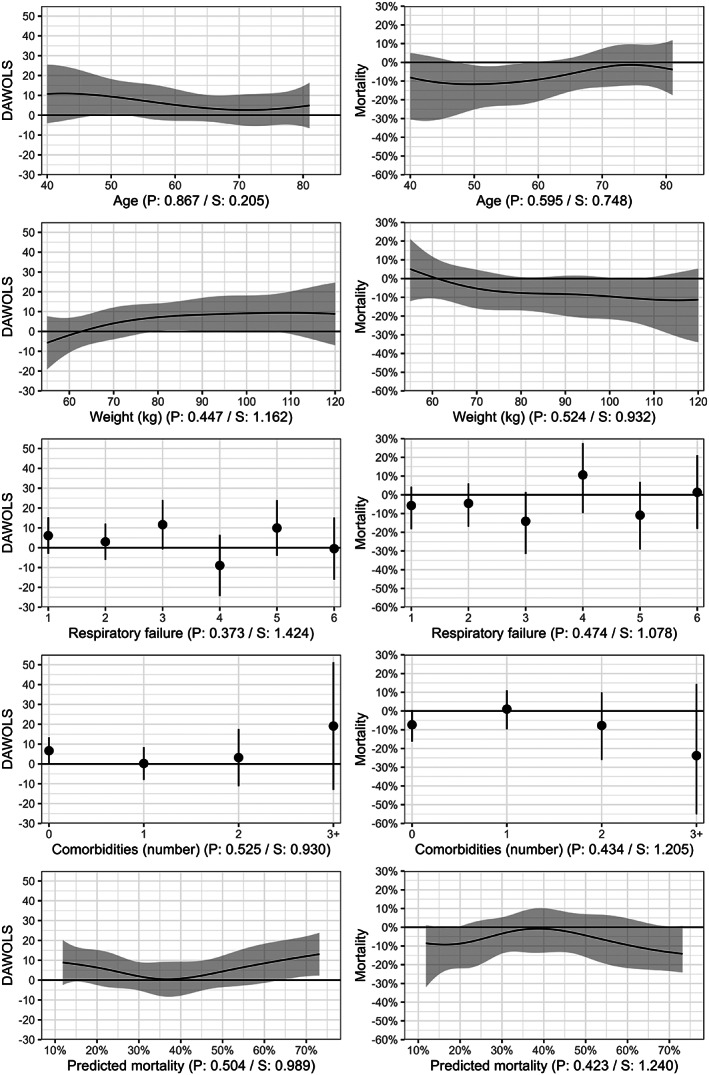
Between‐group differences in outcomes according to various baseline characteristics. Differences in days alive without life support (DAWOLS) and mortality at Day 90 with 95% confidence intervals according to the variables assessed at baseline (including predicted risks of mortality at Day 90 using the internal prediction model) with 95% confidence intervals. Values are presented as the treatment effects of 12‐mg dexamethasone, that is, positive differences indicate higher values in the 12‐mg group and vice versa. For both outcomes, predicted values and 95% confidence intervals are truncated at the lowest/highest possible values (0/90 days and 0/100%, respectively). The *p* and *S* values from the likelihood ratio tests assessing evidence in favour of heterogeneous treatment effects are displayed below each plot. For the continuous variables, predicted differences are only displayed for the central 90% of values in the data due to the large uncertainty at the extreme values with limited data. Figure S5 displays predicted differences across all observed values in the datasets as specified in the statistical analysis plan.^15^

Estimated treatment effects for both outcomes according to the variables assessed are presented in Figure 4 (and Figure S5), which shows the same patterns as Figures 1, 2, 3.

### 3.3 Additional analyses added during peer review

Additional descriptive data and results from analyses added during peer review are presented in the supplement (Table S1 and Figures S6–S7); in brief, there was no strong evidence of HTE, although analyses according to PaO_2_/FiO_2_‐ratios in those on closed systems suggested benefit with 12 mg in most patients, while 6 mg seemed preferable in those with lowest PaO_2_/FiO_2_‐ratios despite substantial uncertainty.

## 4 DISCUSSION

In this post hoc exploratory sub‐study of the COVID STEROID 2 trial, we found no strong evidence for substantial HTE with higher (12 mg) versus lower (6 mg) doses of dexamethasone on days alive without life support or mortality at Day 90 in patients with COVID‐19 and severe hypoxaemia. All *S* values were <1.43 meaning that if there are truly no differences, then observing these results is less surprising than obtaining two heads in a row using a fair coin. While these results provide no strong evidence for HTE, they cannot firmly rule it out either.

We previously hypothesised that higher doses of dexamethasone may be more beneficial in younger patients,^17^ but these results provide no meaningful support for this hypothesis. Others have hypothesised that relatively higher doses may be required in obese patients to avoid underdosing;^26^ while these results do not provide any strong evidence for that hypothesis either, point estimates did point in that direction. Interestingly, in a previous prospective meta‐analysis assessing the effects of systemic corticosteroids, the effects of steroids on mortality seemed to be higher in patients not on invasive mechanical ventilation than in those mechanically ventilated, although the former group only included 144 patients.^2^ We found no firm evidence of HTE according to our categorical scale of respiratory failure; however, some numerical differences in treatment effects were found in patients on closed systems (NIV/CPAP or invasive mechanical ventilation), with possibly reversed or neutral treatment effects in the groups with FiO_2_ above the median value. Similarly, results from the analysis added during peer review assessing HTE according to PaO_2_/FiO_2_‐ratios in those on closed systems were mostly compatible with reversed treatment effects (i.e., preferring 6 mg) in patients with the lowest PaO_2_/FiO_2_‐ratios. A similar signal was not found in those on open systems according to PaO_2_/oxygen flow‐ratios. In keeping with the aforementioned prospective meta‐analysis,^2^ these results could suggest that increased immunosuppression (i.e., higher doses of dexamethasone) provide additional benefits early in the disease course, while this may not be the case later when the disease has progressed. Due to the high uncertainty and post hoc nature of this exploratory study, all these findings should be interpreted very cautiously and need confirmation in subsequent studies.

Assessing HTE according to illness severity defined as the risk of poor outcomes has been recommended,^8^, ^27^ and patients at higher risk of a poor outcome may be hypothesised to have larger beneficial effects of the treatment. We assessed HTE both according to the cumulated number of comorbidities and using a risk modelling approach using an internal prediction model,^8^, ^27^ but found no firm evidence of HTE according to these variables. Thus, it seems that the treatment effects are relatively similar independent of comorbidity burdens and risk of a poor outcome, at least in patients with COVID‐19 and severe hypoxaemia.

### 4.1 Strengths and limitations

This study comes with several strengths, including the overall strengths of the COVID STEROID 2 trial, that is, a relatively large, international pragmatic trial with blinding and limited missing data.^4^ In addition, the strengths of this study include the analysis plan, which was written and made publicly available before the analyses were conducted.^4^, ^15^ Further, we conducted analyses of HTE without dichotomisation of variables (and concomitant loss of information)^14^ and with interpretation of the evidence on the continuous scale without dichotomisation according to *p*‐value thresholds.^28^ Finally, we assessed HTE according to multiple relevant baseline variables, including the overall risk of a poor outcome (as recommended), and the cumulated comorbidity burden, which may better reflect clinical reality than assessing HTE according to individual variables.^8^, ^9^

The study also has limitations, including those general to the COVID STEROID 2 trial, that is, the evolving pandemic and changes in care during and after the trial (i.e., recommendations in favour of interleukin‐6 receptor antagonists introduced after randomisation concluded^3^), and limited power for some analyses.^4^ Moreover, this was a post hoc exploratory study, and despite public registration of the statistical analysis plan prior to the conduct of the analyses, this was done after the primary trial results were known. Consequently, these results should be interpreted cautiously and as hypothesis generating only. Second, to simplify the analyses, we did not adjust for the stratification variables; however, as the results were similar for both outcomes assessed here in the primary adjusted and unadjusted analyses,^4^ this is unlikely to have had any substantial influence on our results. Third, comorbidities were selected according to availability and prevalence in the trial and weighted equally in the analyses according to the number of comorbidities, although some may increase the risk of poor outcomes more than others. Yet, this limitation is mitigated by their inclusion in the internal prediction model. Fourth, our categorisation of respiratory failure is somewhat arbitrary, data‐driven, and specific to this study. The PaO_2_/FiO_2_‐ratio might have been a better measure of respiratory failure, but unfortunately, data were not available to calculate this for patients on open systems,^4^, ^17^ which was the case for slightly more than 50% of the included patients at baseline. However, an additional analysis according to PaO_2_/FiO_2_‐ratios in those on closed systems added during peer review seemed to support the results from the planned analysis of respiratory failure categories. Fifth, we used an internal prediction model as data for external, previously developed prediction models were not registered in the trial. The internal prediction model was developed in the control group only as we knew that mortality rates at Day 90 seemed to differ between groups.^4^, ^5^, ^15^ This approach may come with a risk of potentially exaggerating interactions;^27^ however, as no strong evidence for HTE according to predicted mortality risks was found, this was not an issue here. Finally, while we found no firm evidence of HTE according to the variables assessed, we cannot exclude that it exists and was merely not found due to limited power.

## 5 CONCLUSIONS

In conclusion, we found no convincingly strong evidence for substantial HTE with higher (12 mg) versus lower (6 mg) doses of dexamethasone on days alive without life support or mortality at Day 90 in patients with COVID‐19 and severe hypoxaemia according to age, weight, number of comorbidities, category of respiratory failure or predicted risks of mortality.

## AUTHOR CONTRIBUTIONS

This exploratory, post hoc study was conceived and planned by Anders Granholm, Marie Warrer Munch and Anders Perner. Anders Granholm conducted all analyses presented in this manuscript and wrote the first draft, which was critically revised by all authors. Marie Warrer Munch was the coordinating investigator of the COVID STEROID 2 trial, and Anders Perner was the trial sponsor. All authors contributed to the design and/or conduct of the trial. Detailed author contributions for the full COVID STEROID 2 trial were presented in the primary trial report.^4^

## FUNDING INFORMATION

The COVID STEROID 2 trial was funded by Novo Nordisk Foundation and the Research Council of Rigshospitalet. The funders had no role in the design, conduct, analyses or reporting of the trial or this secondary study.

## CONFLICT OF INTEREST

Anders Granholm, Marie Warrer Munch, Morten Hylander Møller and Anders Perner are affiliated with the Department of Intensive Care at Rigshospitalet—Copenhagen University Hospital, which has received funding for other projects from the Novo Nordisk Foundation, Sygeforsikringen ‘danmark’, Pfizer and Fresenius Kabi, and conducts contract research for AM‐Pharma. Vivekanand Jha has received grant funding from GSK, Baxter Healthcare and Biocon and has received honoraria from Bayer, AstraZeneca, Boeringer Ingelheim, NephroPlus and Zydus Cadilla, under the policy of all honoraria being paid to the organisation. Stephan M. Jakob reports that the Department of Intensive Care Medicine, University Hospital Bern, has or has had in the past, research and development/consulting contracts with Edwards Lifesciences Services GmbH, Phagenesis Limited, Nestlé and Cytel Inc. The money was paid into a departmental fund, Dr Jakob did not receive any financial gain. The Department of Intensive Care Medicine, University Hospital Bern has received in the past unrestricted educational grants from the following organisations for organising bi‐annual postgraduate courses in the fields of critical care ultrasound, management of ECMO and mechanical ventilation: Pierre Fabre Pharma AG (formerly known as RobaPharm), Pfizer AG, Bard Medica S.A., Abbott AG, Anandic Medical Systems, PanGas AG Healthcare, Orion Pharma, Bracco, Edwards Lifesciences AG, Hamilton Medical AG, Fresenius Kabi (Schweiz) AG, Getinge Group Maquet AG, Dräger Schweiz AG and Teleflex Medical GmbH.

## Supporting information


**Appendix S1:** Supporting Information
