# Supplementary material for: Heterogeneous treatment effects of dexamethasone 12 mg versus 6 mg in patients with COVID‐19 and severe hypoxaemia—Post hoc exploratory analyses of the COVID STEROID 2 trial
Source: Acta Anaesthesiol Scand. 2022 Nov 8;67(2):195–205. doi: 10.1111/aas.14167 (PMC9874464; doi:10.1111/aas.14167)
Supplement: Supplementary file 1 — Appendix S1: Supporting Information [file AAS-67--s001.pdf]

Heterogenous treatment effects of dexamethasone 12 mg vs. 6 mg in patients with COVID-19 and severe hypoxaemia – *post hoc* exploratory analyses of the COVID STEROID 2 trial

Supplement

Table of contents

Figure S1: Days alive without life support at day 90 according to treatment allocation and continuous baseline characteristics without truncation 2
Figure S2: Mortality at day 90 according to treatment allocation and continuous baseline characteristics without truncation 3
Internal prediction model 4
Figure S3: Calibration plot for the internal prediction model for 90-day mortality 5
Figure S4: Days alive without life support and mortality at day 90 according to treatment allocation and predicted mortality risk without truncation 6
Figure S5: Between-group differences in outcomes according to various continuous baseline characteristics without truncation 7
Table S1: Additional descriptive baseline data 9
Figure S6: Days alive without life support and mortality at day 90 according to treatment allocation and oxygenation data 10
Figure S7: Between-group differences in outcomes according to oxygenation data 11
STROBE checklist 12
References 15

Figure S1: Days alive without life support at day 90 according to treatment allocation and continuous baseline characteristics without truncation

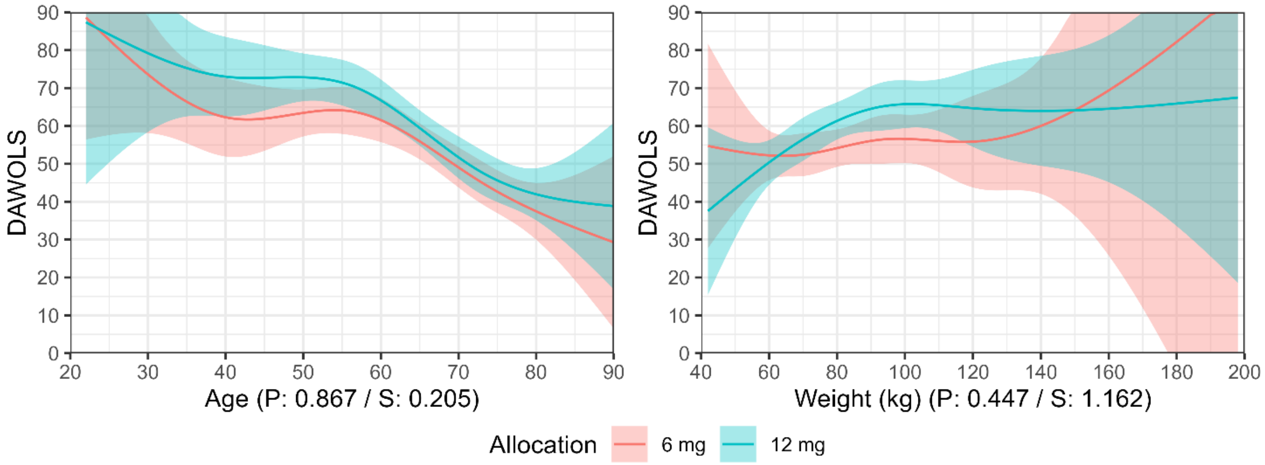

Expected mean number of days alive without life support (DAWOLS) with 95% confidence intervals according to two continuous baseline variables (age and weight, as described in the methods section of the main text) according to the models fit. Predicted values and 95% confidence intervals are truncated at the lowest/highest possible values (0/90 days). P-values and S-values from the likelihood ratio tests assessing evidence in favour of heterogeneous treatment effects are displayed below each plot.

Predictions are displayed for all values in the data as specified in the statistical analysis plan,[1] but there is large uncertainty at the extreme values due to limited data. Predictions for the central 90% of values only are presented in **Figure 1** in the main text.

Figure S2: Mortality at day 90 according to treatment allocation and continuous baseline characteristics without truncation

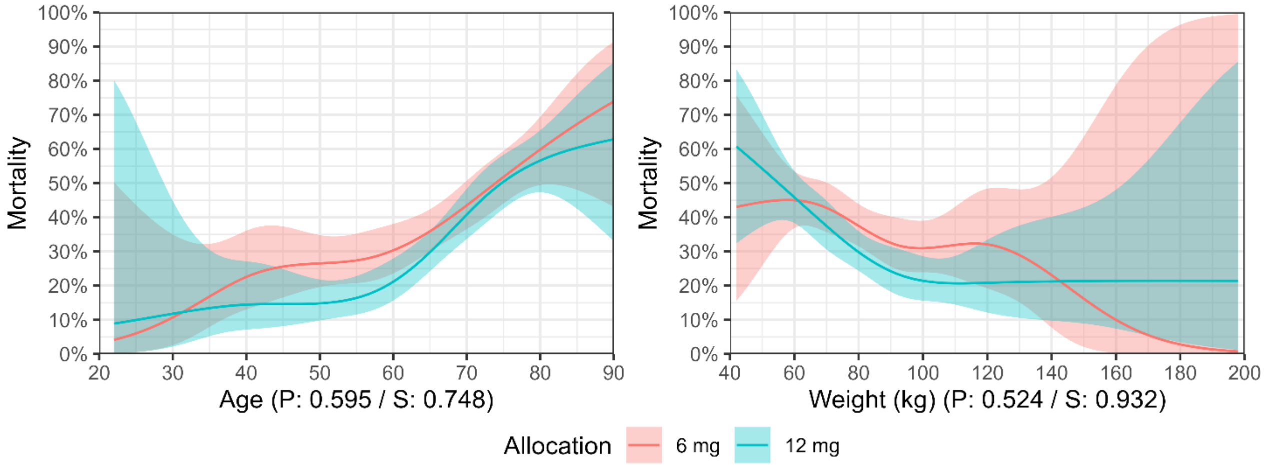

Expected mortality ratios with 95% confidence intervals according to two continuous baseline variables (age and weight, as described in the methods section of the main text) according to the models fit. P-values and S-values from the likelihood ratio tests assessing evidence in favour of heterogeneous treatment effects are displayed below each plot.

Predictions are displayed for all values in the data as specified in the statistical analysis plan,[1] but there is large uncertainty at the extreme values due to limited data. Predictions for the central 90% of values only are presented in **Figure 2** in the main text.

Internal prediction model

The internal prediction model for 90-day mortality in the control group[1] had the following form with *logit(p)* corresponding to the predicted probability on the logit scale:

$$logit(p) = -3.30228 - 0.08580 * (age\ [years]/100)^{-2} + 3.14149 * (age\ [years]/100)^{3} + 1.54123 * (weight\ [kg]/100)^{-2} + 1.40017 * ((weight\ [kg]/100)^{-2} * log(weight\ [kg]/100)) + 0.35134 * NIV\ or\ CPAP + 0.80835 * invasive\ mechanical\ ventilation - 0.12557 * ischaemic\ heart\ disease\ or\ heart\ failure + 1.08762 * immunosuppression + 0.10269 * chronic\ obstructive\ pulmonary\ disease + 0.11773 * diabetes\ mellitus - 0.23646 * lactate\ [mmol/L] + 0.03265 * (lactate\ [mmol/L])^{2} + 0.41006 * use\ of\ vasopressors\ or\ inotropes + 1.20796 * limitations\ in\ care$$

Predicted 90-day mortality risk can thus be calculated as:

$$Predicted\ probability = exp(logit(p)) / (1 + exp(logit(p))$$

Abbreviations: CPAP: continuous positive airway pressure; NIV: non-invasive ventilation.

Figure S3: Calibration plot for the internal prediction model for 90-day mortality

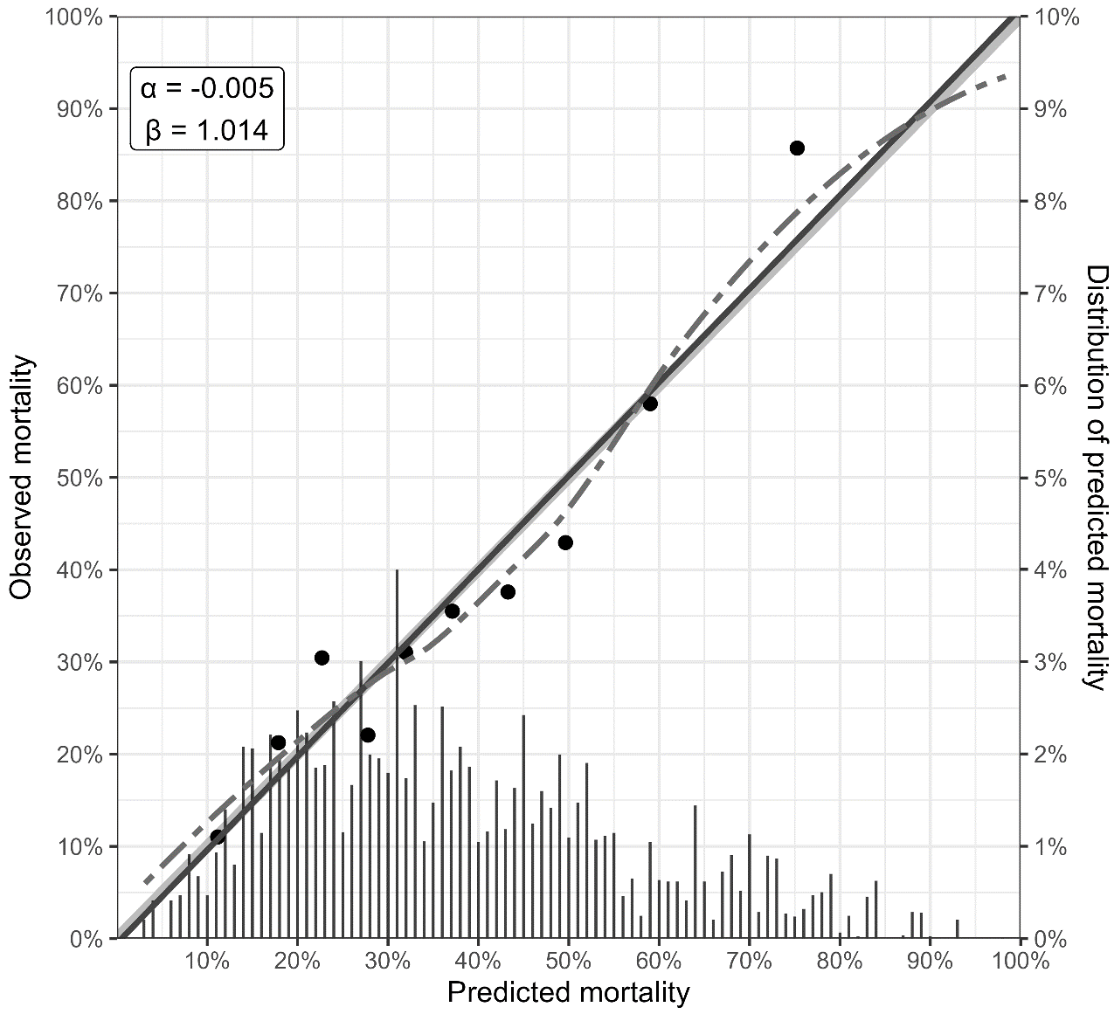

Calibration plot showing apparent, internal calibration of the internal prediction model for 90-day mortality developed and assessed in the control group only. The plots display predicted versus observed mortality with each point corresponding to tenths of the data grouped according to increasing predicted mortality. The black, full line is the line of identity (predicted = observed mortality, i.e., perfect calibration); the grey, full line corresponds to a linear regression model of observed mortality (dependent variable) according to predicted mortality (independent variable), with the intercept α and slope β displayed on the plot; the dashed, grey line represents a *loess* smoothing function between the same values. Finally, the distribution of predicted mortality values is displayed as bars.

Figure S4: Days alive without life support and mortality at day 90 according to treatment allocation and predicted mortality risk without truncation

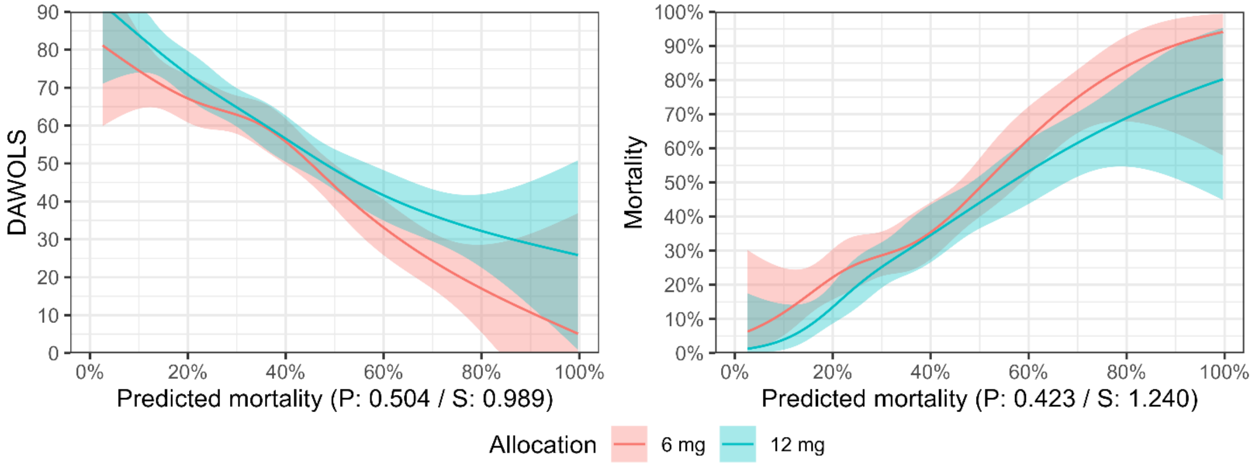

Expected mean number of days alive without life support (DAWOLS) and risk of mortality at day 90 with 95% confidence intervals according to the predicted risks of mortality at day 90 using the internal prediction model. For DAWOLS, predicted values and 95% confidence intervals are truncated at the lowest/highest possible values (0/90 days). P-values and S-values from the likelihood ratio tests assessing evidence in favour of heterogeneous treatment effects are displayed below each plot.
Predictions are displayed for all values in the data as specified in the statistical analysis plan,[1] but there is large uncertainty at the extreme values due to limited data. Predictions for the central 90% of values only are presented in **Figure 3** in the main text.

Figure S5: Between-group differences in outcomes according to various continuous baseline characteristics without truncation

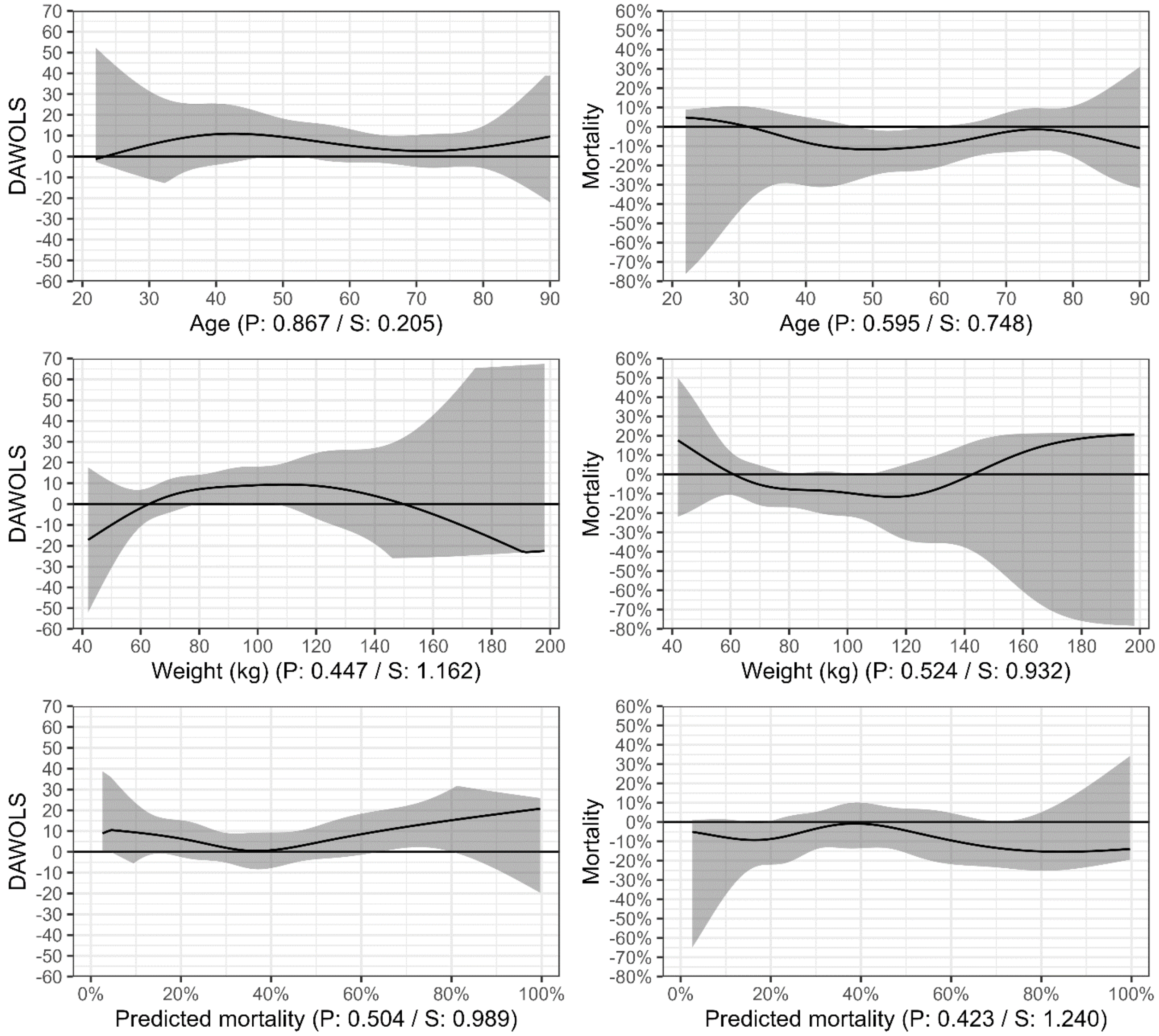

Differences in days alive without life support (DAWOLS) and mortality at day 90 with 95% confidence intervals according to the continuous variables assessed at baseline (including predicted risks of mortality at day 90 using the internal prediction model) with 95% confidence intervals. Values are presented as the treatment effects of 12 mg dexamethasone, i.e., positive difference indicate higher values in the 12 mg group and vice versa. For both outcomes, predicted values and 95% confidence intervals are truncated at the lowest/highest possible vales (0/90 days and 0/100%, respectively). P-values and S-values from the likelihood ratio tests assessing evidence in favour of heterogeneous treatment effects are displayed below each plot.
Predicted differences are only displayed for all values in the data as specified in the statistical analysis plan,[1] but there is large uncertainty at the extreme values due to limited data, and due to

this uncertainty and truncation, point estimates and limits of the 95% confidence intervals are similar in some parts of the plots. The central 90% of values is presented in **Figure 4** in the main text.

Table S1: Additional descriptive baseline data

**Variable**	**12 mg (n = 497)**	**6 mg (n = 485)**
PaO_2 (kPa)[a]	9.6 (8.2 - 11.6) [3.0 - 53.1]	9.4 (8.1 - 11.0) [3.2 - 45.9]
PaO_2/FiO_2-ratio (kPa, in patients on closed system only)[b]	18.0 (12.9 - 24.6) [6.6 - 132.8]	17.3 (11.8 - 23.4) [5.4 - 140.3]
PaO_2/oxygen flow rate (kPa/L/min, in patients on open system only)[c]	0.4 (0.3 - 0.6) [0.1 - 3.3]	0.4 (0.2 - 0.6) [0.1 - 1.8]

Additional descriptive baseline data added during peer review; data are presented as medians (interquartile ranges) [full ranges].

Abbreviations: FiO_2: fraction of inspired oxygen; kPa: kilopascals; PaO_2: partial pressure of oxygen in arterial blood.

[a] Missing in 51 (5.2%) of all patients.

[b] Missing in 27 (6.0%) of all patients on closed systems.

[c] Missing in 28 (5.3%) of all patients on open systems.

Figure S6: Days alive without life support and mortality at day 90 according to treatment allocation and oxygenation data

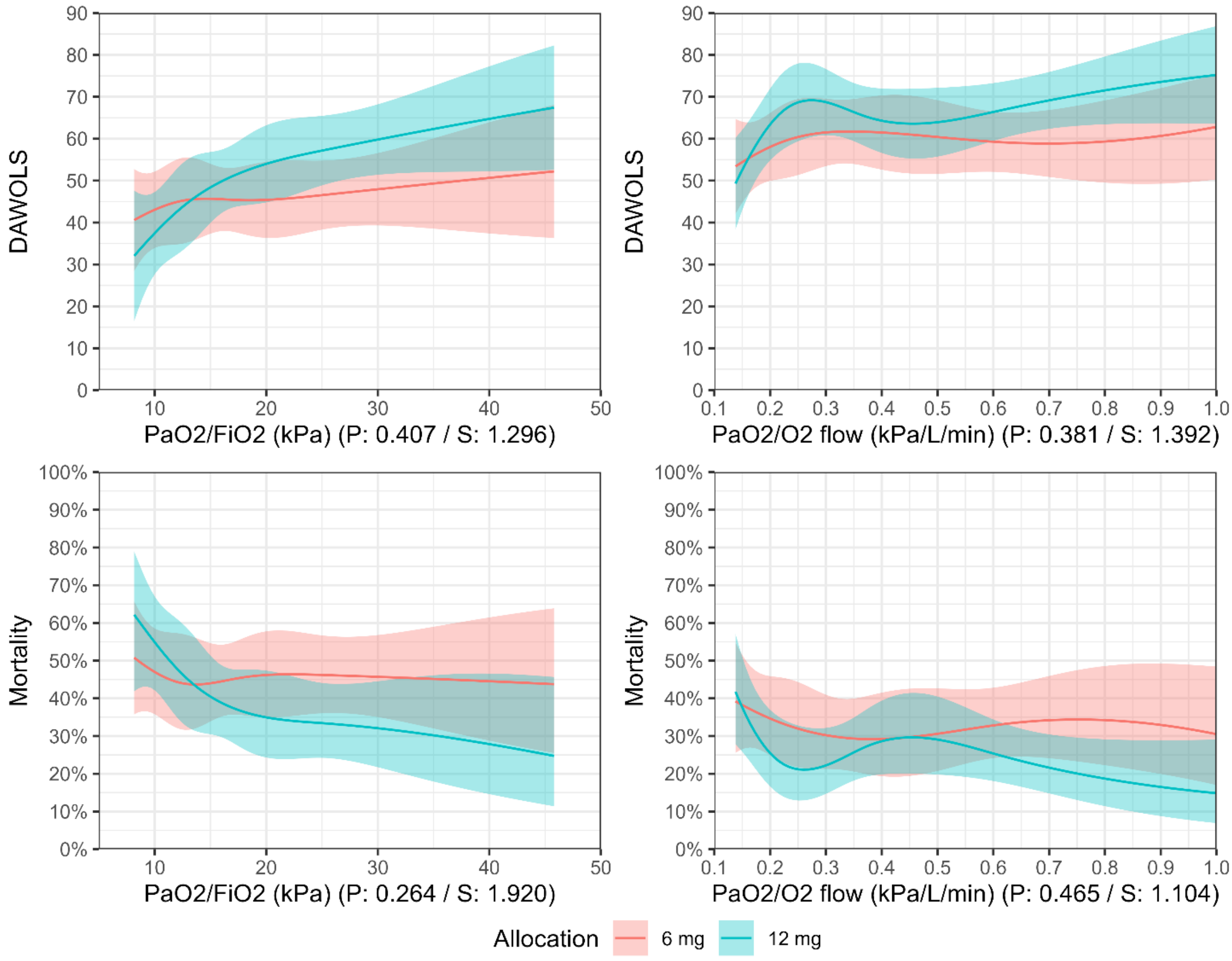

Expected mean number of days alive without life support (DAWOLS) and risk of mortality at day 90 with 95% confidence intervals according to PaO_2/FiO_2-ratios in patients on closed systems only and PiO_2/O_2 flow-ratios in patients on open systems only. For DAWOLS, predicted values and 95% confidence intervals are truncated at the lowest/highest possible values (0/90 days). P-values and S-values from the likelihood ratio tests assessing evidence in favour of heterogeneous treatment effects are displayed below each plot.

For these continuous variables, predictions are only displayed for the central 90% of values in the data due to large uncertainty at the extreme values with limited data. These analyses were added during peer review and conducted using complete cases only.

Abbreviations: FiO_2: fraction of inspired oxygen; kPa: kilopascals; O_2: oxygen; PaO_2: partial pressure of oxygen in arterial blood.

Figure S7: Between-group differences in outcomes according to oxygenation data

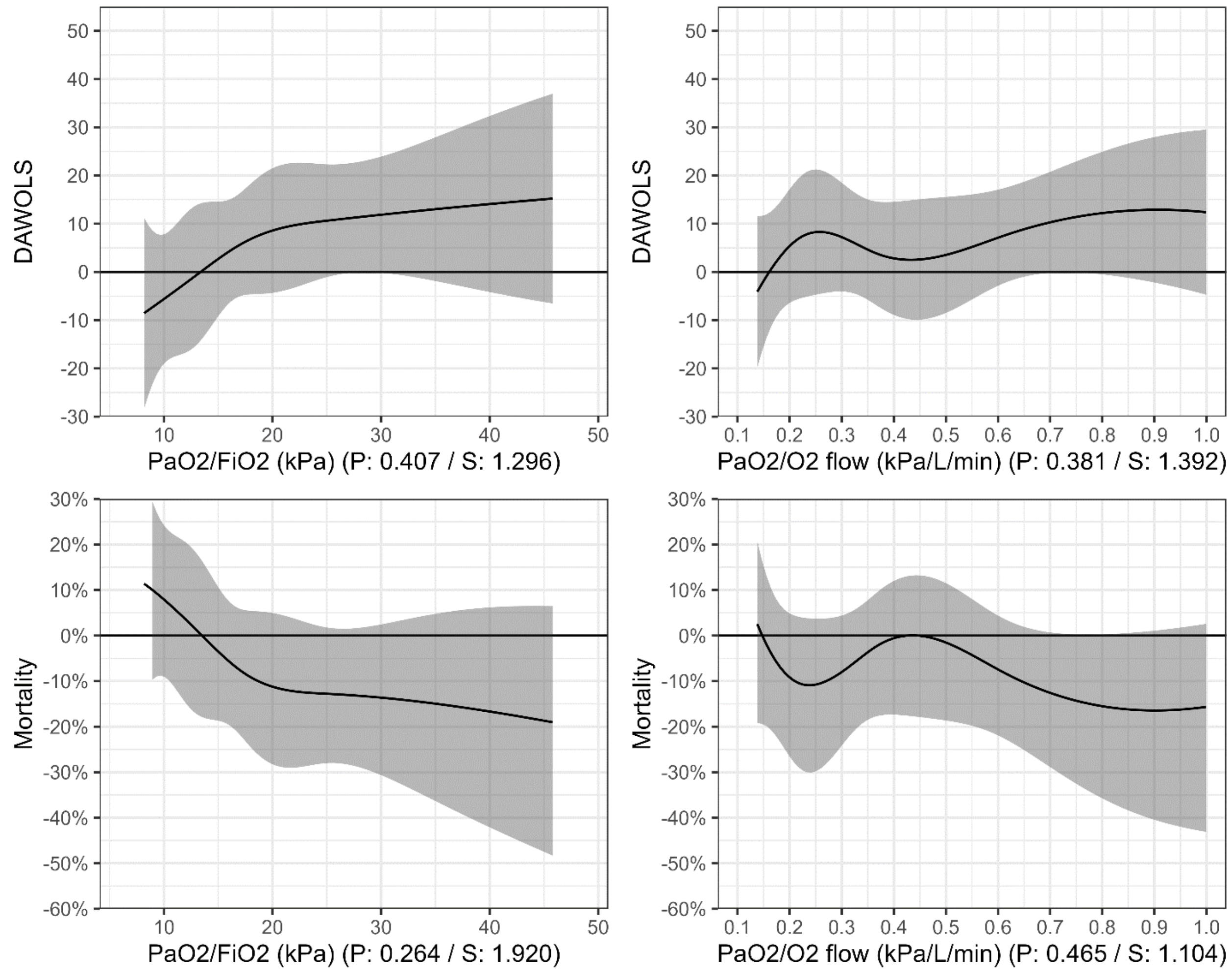

Differences in days alive without life support (DAWOLS) and mortality at day 90 with 95% confidence intervals to PaO_2/FiO_2-ratios in patients on closed systems only and PiO_2/O_2 flow-ratios in patients on open systems only with 95% confidence intervals. Values are presented as the treatment effects of 12 mg dexamethasone, i.e., positive difference indicate higher values in the 12 mg group and vice versa. For both outcomes, predicted values and 95% confidence intervals are truncated at the lowest/highest possible vales (0/90 days and 0/100%, respectively). P-values and S-values from the likelihood ratio tests assessing evidence in favour of heterogeneous treatment effects are displayed below each plot.
For these continuous variables, predicted differences are only displayed for the central 90% of values in the data due to large uncertainty at the extreme values with limited data. These analyses were added during peer review and conducted using complete cases only.

Abbreviations: FiO_2: fraction of inspired oxygen; kPa: kilopascals; O_2: oxygen; PaO_2: partial pressure of oxygen in arterial blood.

STROBE checklist

Completed Strengthening the Reporting of Observational Studies in Epidemiology (STROBE) statement checklist[2] for this manuscript. Additional details can be found in the primary trial protocol and the primary trial report.[1,3,4] This manuscript is reported according to STROBE despite the randomised design to avoid unnecessary duplication of items, as STROBE covers all applicable items not specified in the primary trial report adhering to the Consolidated Standards of Reporting Trials (CONSORT) statement.[2,4,5]

STROBE Statement—Checklist of items that should be included in reports of ***cohort studies***

	Item No	Recommendation
**Title and abstract**	1	(*a*) Indicate the study's design with a commonly used term in the title or the abstract – 2
		(*b*) Provide in the abstract an informative and balanced summary of what was done and what was found – 2
**Introduction**		
Background/rationale	2	Explain the scientific background and rationale for the investigation being reported – 3
Objectives	3	State specific objectives, including any prespecified hypotheses – 3
**Methods**		
Study design	4	Present key elements of study design early in the paper – 4
Setting	5	Describe the setting, locations, and relevant dates, including periods of recruitment, exposure, follow-up, and data collection – 4
Participants	6	(*a*) Give the eligibility criteria, and the sources and methods of selection of participants. Describe methods of follow-up – 4, primary trial report and protocol
		(*b*) For matched studies, give matching criteria and number of exposed and unexposed – not applicable
Variables	7	Clearly define all outcomes, exposures, predictors, potential confounders, and effect modifiers. Give diagnostic criteria, if applicable – 4-5, primary trial report and protocol
Data sources/ measurement	8*	For each variable of interest, give sources of data and details of methods of assessment (measurement). Describe comparability of assessment methods if there is more than one group – 4-5, primary trial report and protocol
Bias	9	Describe any efforts to address potential sources of bias – 4
Study size	10	Explain how the study size was arrived at – 4, primary trial report and protocol

Quantitative variables	11	Explain how quantitative variables were handled in the analyses. If applicable, describe which groupings were chosen and why – 4-6
Statistical methods	12	(*a*) Describe all statistical methods, including those used to control for confounding – 4-7
		(*b*) Describe any methods used to examine subgroups and interactions – 5-7
		(*c*) Explain how missing data were addressed – 6-7
		(*d*) If applicable, explain how loss to follow-up was addressed – 6-7
		(*e*) Describe any sensitivity analyses – not applicable
**Results**		
Participants	13*	(a) Report numbers of individuals at each stage of study—eg numbers potentially eligible, examined for eligibility, confirmed eligible, included in the study, completing follow-up, and analysed – 8-9, primary trial report
		(b) Give reasons for non-participation at each stage – primary trial report
		(c) Consider use of a flow diagram – primary trial report
Descriptive data	14*	(a) Give characteristics of study participants (eg demographic, clinical, social) and information on exposures and potential confounders – Table 1, primary trial report, supplement
		(b) Indicate number of participants with missing data for each variable of interest – Table 1, supplement
		(c) Summarise follow-up time (eg, average and total amount) – 4
Outcome data	15*	Report numbers of outcome events or summary measures over time – Table 1, primary trial report
Main results	16	(*a*) Give unadjusted estimates and, if applicable, confounder-adjusted estimates and their precision (eg, 95% confidence interval). Make clear which confounders were adjusted for and why they were included – 8, Figures, supplement
		(*b*) Report category boundaries when continuous variables were categorized – not applicable/5
		(*c*) If relevant, consider translating estimates of relative risk into absolute risk for a meaningful time period – not applicable
Other analyses	17	Report other analyses done—eg analyses of subgroups and interactions, and sensitivity analyses – 8-9, supplement
**Discussion**		
Key results	18	Summarise key results with reference to study objectives – 10
Limitations	19	Discuss limitations of the study, taking into account sources of potential bias or imprecision. Discuss both direction and magnitude of any potential bias – 10-11

Interpretation	20	Give a cautious overall interpretation of results considering objectives, limitations, multiplicity of analyses, results from similar studies, and other relevant evidence – 10-11
Generalisability	21	Discuss the generalisability (external validity) of the study results – 10
**Other information**		
Funding	22	Give the source of funding and the role of the funders for the present study and, if applicable, for the original study on which the present article is based – 12

*Give information separately for exposed and unexposed groups.

References

1. Granholm A, Munch MW, Perner A. COVID STEROID 2 trial: outline for a secondary post-hoc study assessing heterogeneous treatment effects on the continuous scale. *OSF Registries* 2022. https://doi.org/10.17605/OSF.IO/523KH.

2. von Elm E, Altman DG, Egger M, Pocock SJ, Gøtzsche PC, Vandenbroucke JP. The Strengthening the Reporting of Observational Studies in Epidemiology (STROBE) statement: Guidelines for reporting observational studies. *J Clin Epidemiol* 2008;61:344–349.

3. Munch MW, Granholm A, Myatra SN, et al. Higher vs. Lower Doses of Dexamethasone in Patients with COVID-19 and Severe Hypoxia (COVID STEROID 2) trial: protocol and statistical analysis plan. *Acta Anaesthesiol Scand* 2021;65:834-845.

4. COVID STEROID 2 Trial Group. Effect of 12 mg vs 6 mg of Dexamethasone on the Number of Days Alive Without Life Support in Adults With COVID-19 and Severe Hypoxemia. *JAMA* 2021;326:1807-1817.

5. Schulz KF, Altman DG, Moher D, CONSORT Group. CONSORT 2010 statement: updated guidelines for reporting parallel group randomised trials. *BMJ* 2010;340:c332.
